# Teaching Modalities for Hyperbaric Oxygen Therapy Education: A Systematic Review and Narrative Synthesis

**DOI:** 10.3390/healthcare14213661

**Published:** 2026-10-28

**Authors:** Siya Pei, Yongwen Yang, Xuyuan Kuang

**Affiliations:** 1 Department of Hyperbaric Oxygen, Xiangya Hospital Central South University, Changsha 410008, China; 2 Department of Hyperbaric Oxygen, National Regional Center for Neurological Diseases, Jiangxi Hospital of Xiangya Hospital, Central South University, Nanchang 330006, China; 3 National Clinical Research Center for Geriatric Diseases (Xiangya Hospital), Changsha 410008, China; 4 Department of Clinical Laboratory, Xiangya Hospital, Central South University, Changsha 410008, China

**Keywords:** hyperbaric oxygen therapy, medical education, simulation-based education, blended learning, systematic review, narrative synthesis, technology-enhanced learning

## Abstract

Objective: To systematically identify and synthesise the available evidence on teaching modalities for hyperbaric oxygen therapy (HBOT) education across all learner levels. Methods: We searched PubMed, Scopus, Web of Science, CNKI, Wanfang, SinoMed, EMBASE, and the Cochrane Library from inception to 15 June 2026 for studies evaluating any HBOT teaching or training intervention against traditional instruction or another comparator, with knowledge, skills, or learner satisfaction as outcomes. Risk of bias was assessed using the Cochrane RoB 2 tool for randomised trials, ROBINS-I for comparative non-randomised studies, and MMAT/JBI checklists for descriptive and survey designs. Because of marked design heterogeneity, findings were synthesised narratively following the SWiM guideline. An exploratory synthesis of extractable effect sizes was undertaken to characterise the direction and magnitude of reported effects. The protocol was retrospectively registered in PROSPERO (CRD420261462169). Results: Thirty studies met the inclusion criteria (nine English-language, 21 Chinese-language). The evidence base was dominated by single-group pre–post evaluations, descriptive curriculum reports, and cross-sectional surveys; only four randomised controlled trials were identified. Technology-enhanced modalities—simulation-based education, blended/online learning, and Web-based simulation—were generally associated with promising improvements in knowledge and skills, although most studies lacked an active comparator. The exploratory synthesis identified only two studies with extractable knowledge outcome effect sizes, both implausibly large (g > 2.0), likely reflecting small-sample bias, absence of blinding, and practice effects. Conclusions: The available evidence suggests that technology-enhanced teaching modalities are promising for HBOT education, but the field lacks methodologically rigorous comparative studies and no study yet demonstrates consistent superiority over traditional didactic teaching. The anomalously large effect sizes in the few quantitative studies signal a high risk of bias and an urgent need for adequately powered randomised trials with blinded outcome assessment, standardised outcome measures, and a consensus-derived competency framework.

## 1. Introduction

Hyperbaric oxygen therapy (HBOT) delivers 100% oxygen at pressures above one atmosphere absolute and is indicated for decompression illness, carbon monoxide poisoning, necrotising soft-tissue infections, compromised grafts and flaps, and radiation tissue injury, among other conditions [1,2]. Despite its established clinical role, HBOT remains among the least formally taught disciplines in undergraduate and postgraduate medical curricula worldwide, a finding supported by cross-sectional surveys of knowledge and curricular exposure among medical students and physicians [3,4,5,6]. Disciplines that most commonly require this education include emergency medicine, critical care, diving and undersea medicine, wound care, and rehabilitation medicine, where practitioners must recognise and manage indications for HBOT and its complications [7,8].

The educational challenge in HBOT is distinctive. Learners must master hyperbaric physiology—gas laws, oxygen toxicity thresholds, and bubble dynamics—alongside operational safety protocols and the management of rare but catastrophic events such as central nervous system oxygen toxicity and pneumothorax under pressure. Traditional didactic lectures, historically the default mode of HBOT instruction, are poorly suited to conveying these procedural and crisis management competencies.

Over the past two decades, a shift toward competency-based medical education [9] has prompted evaluation of diverse HBOT teaching approaches, including simulation-based education [10,11,12], in situ simulation [13], Web-based chamber simulators [14], blended and WeChat-based learning [15,16], problem-based and case-based learning [17,18], experiential teaching [19], virtual reality in adjacent aerospace medicine settings [20], and scenario-based emergency preparedness drills [21]. International training and credentialling frameworks further specify the competencies required of HBOT personnel [22,23,24,25]. A prior systematic review by Boet et al. (2019) identified no comparative studies of simulation in HBOT education and concluded that the evidence base was effectively absent [11].

To our knowledge, no review has comprehensively synthesised the effectiveness of all available HBOT teaching modalities across learner levels, nor incorporated the growing body of Chinese-language technology-enhanced educational research. This review therefore aims to (1) systematically identify and appraise the comparative effectiveness of HBOT teaching modalities, (2) characterise the design and quality of the existing evidence base, and (3) provide best-evidence recommendations to guide curriculum development and future research in this under-served educational domain. Given the anticipated scarcity of comparative studies, this review was designed as an evidence mapping review: it systematically maps all published HBOT teaching interventions and educational evaluations, regardless of study design, to characterise the breadth and quality of the evidence base, with a nested subset of studies offering extractable effect estimates analysed for the direction and magnitude of effect.

## 2. Methods

### 2.1. Protocol and Registration

This review was conducted in accordance with the Preferred Reporting Items for Systematic Reviews and Meta-Analyses (PRISMA) 2020 statement [26]. The review protocol was registered in the International Prospective Register of Systematic Reviews (PROSPERO; CRD420261462169) on 29 July 2026; because the literature search was completed on 15 June 2026, this registration was retrospective. Although a meta-analysis was originally planned, marked clinical and methodological heterogeneity and the scarcity of extractable effect sizes precluded any pooled estimate, and findings were therefore synthesised narratively following the Synthesis Without Meta-analysis (SWiM) guideline [27].

### 2.2. Eligibility Criteria

We included studies meeting the following Population, Intervention, Comparator, Outcome, Study design (PICOS) criteria: (P) learners at any level—undergraduate medical students, postgraduate residents/fellows, nurses, and allied health professionals—receiving HBOT education or training; (I) any teaching intervention (simulation, blended/online, problem-based/case-based learning, experiential, or technology-enhanced approaches); (C) traditional didactic instruction or no active comparator; (O) knowledge acquisition, practical skills, or learner satisfaction; and (S) any comparative, pre–post, or descriptive design, including curriculum evaluations and training needs surveys. We excluded opinion pieces, conference abstracts without full text, and non-educational studies. Consistent with the evidence mapping design, a small number of studies without a formal comparator were retained where they directly addressed HBOT or closely allied (aerospace/hyperbaric) education and were judged essential to mapping the available approaches; these included a virtual reality study in aerospace medicine [20], a blended learning study for neurology postgraduates [16], and a case-based learning study in hyperbaric neurology [18].

### 2.3. Search Strategy

We searched PubMed, Scopus, Web of Science, EMBASE, the Cochrane Library, CNKI, Wanfang, and SinoMed from inception to 15 June 2026. Reference lists of included studies and relevant reviews were hand-searched; in addition, UHMS annual scientific meeting abstracts (2020–2025) and the tables of contents of Diving and Hyperbaric Medicine (2020–2026) were screened. Search terms combined hyperbaric concepts (“hyperbaric oxygen”, “hyperbaric medicine”, “diving medicine”, “undersea”) with education concepts (“education”, “teaching”, “training”, “simulation”, “curriculum”, “blended learning”, “problem-based learning”, “virtual reality”). Database searching yielded 265 records (PubMed 86, Scopus 74, Web of Science 58, CNKI 47); with Wanfang, SinoMed, EMBASE and the Cochrane Library, the total was 312 records, with 8 additional records identified by hand-searching.

### 2.4. Study Selection and Data Extraction

Two reviewers independently screened titles and abstracts, then assessed full texts against eligibility criteria; disagreements were resolved by consensus. Data were extracted into a standardised form covering study design, learner population, sample size, intervention, comparator, and outcomes.

### 2.5. Risk-of-Bias Assessment

Risk of bias was assessed independently by two reviewers, with disagreements resolved by consensus. The Cochrane Risk of Bias 2 (RoB 2) tool was applied to randomised trials [28]; ROBINS-I (Risk Of Bias In Non-randomised Studies—of Interventions) [29] was reserved for comparative non-randomised studies that targeted an effect estimate. Cross-sectional surveys, descriptive curriculum reports, and needs assessments—designs for which ROBINS-I is not appropriate—were appraised using the Mixed Methods Appraisal Tool (MMAT) or the relevant Joanna Briggs Institute (JBI) critical appraisal checklist. Data extraction and risk-of-bias assessment were both performed in duplicate.

### 2.6. Data Synthesis

Owing to substantial heterogeneity in study design, learner populations, and outcome instruments, findings were synthesised narratively following the Synthesis Without Meta-analysis (SWiM) guideline [27]. As an exploratory synthesis of extractable effect sizes, we computed standardised effect sizes (Hedges’ g or Cohen’s d) for knowledge or procedural-performance outcomes wherever means and standard deviations were extractable, to characterise the direction and magnitude of reported effects; this analysis was explicitly not intended to produce a definitive pooled estimate. Hedges’ g was computed as the standardised mean difference with a small-sample bias correction, and Cohen’s d for paired data as the mean change divided by the standard deviation of the change; where the standard deviation of the change was unavailable, it was estimated from the pre- and post-test standard deviations assuming a correlation of 0.5. All analyses were performed in R version 4.3.2 using R version 4.3.2 and the metafor package (version 4.3-2). Where appropriate, results were interpreted against Miller’s pyramid of clinical competence [30].

## 3. Results

### 3.1. Study Selection

**Figure 1:**
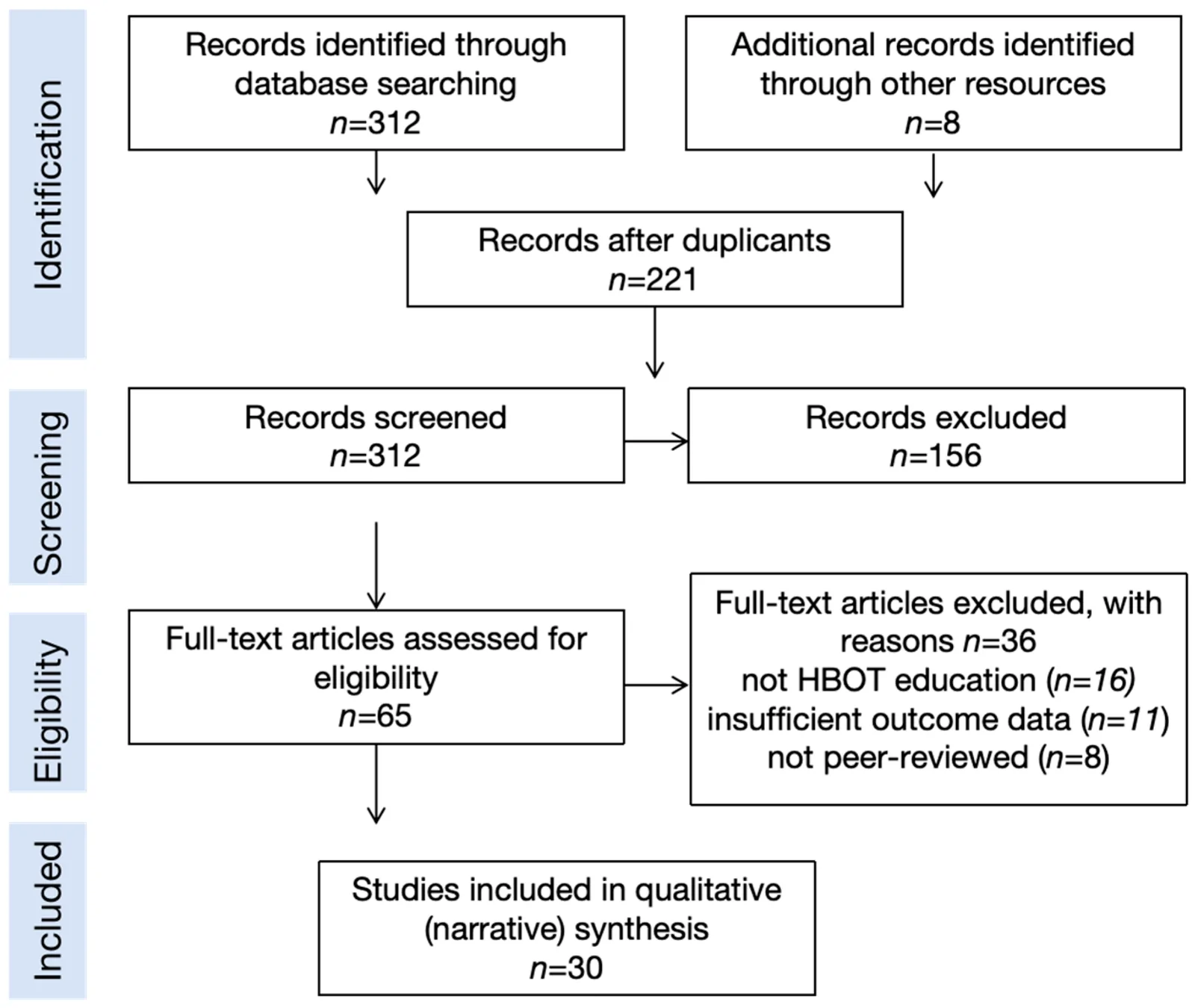
PRISMA 2020 flow diagram of study selection.

Database searching yielded 312 records, with eight additional records identified through reference and grey literature searching. After the removal of 99 duplicates, 221 records were screened, 65 full-text articles were assessed, and 30 studies met the inclusion criteria. Figure 1 presents the PRISMA flow diagram.

### 3.2. Study Characteristics

The 30 included studies were published between 2001 and 2025 and comprised nine English-language and 21 Chinese-language studies. Reported sample sizes ranged from 15 to 240 learners, encompassing undergraduate medical students, residents, postgraduate trainees, nurses, and multidisciplinary teams. The methodological quality was modest: four studies used randomised allocation [17,31,32,33], one used a quasi-experimental design [20], six used single-group pre–post designs [14,15,16,21,34,35], and the remaining nineteen were descriptive curriculum evaluations, training needs surveys, cross-sectional assessments, or a post-course evaluation [3,4,5,6,10,13,18,19,36,37,38,39,40,41,42,43,44,45,46]. Table 1 summarises the characteristics of the included studies and provides the reference number for each included study. Table S2 summarises the included studies by learner level.

### 3.3. Risk of Bias

Among the four randomised trials, risk of bias was predominantly “some concerns”, driven largely by the inherent difficulty of blinding participants and personnel to educational interventions. Comparative non-randomised studies assessed with ROBINS-I were at serious or critical risk of bias owing to uncontrolled confounding and the absence of independent comparison groups. Descriptive and cross-sectional studies, appraised with MMAT/JBI checklists, were generally at high risk of bias or of unclear methodological quality. A per-study, domain-level risk-of-bias assessment for all 30 studies is provided in Supplementary Table S4.

### 3.4. Narrative Synthesis by Teaching Modality

Table S1 presents the narrative synthesis by teaching modality. Multiple studies described simulation-based HBOT education, including a simulation curriculum for central nervous system oxygen toxicity and an intubated patient [10], in situ simulation of underwater and hyperbaric emergencies [13], scenario-based emergency preparedness drills for chamber staff and nurses [21,35], and a Delphi-derived international simulation curriculum [12]. These studies described or suggested improvements in learner engagement, confidence, or emergency-response preparedness, although comparative effectiveness was rarely assessed [10,12,13,21,35].

Web-based and virtual simulation. A pilot study of a Web-based hyperbaric chamber simulator reported large pre–post improvements in procedural proficiency and emergency response; using the reported pre–post data, we calculated a paired Cohen’s d of 2.36 among 27 trainees [14]. A Chinese study applying virtual reality combined with online case-based teaching in aerospace medicine reported significantly higher knowledge scores than traditional instruction in nine of 15 assessment items [20].

Blended and online learning. Chinese studies of WeChat-based blended learning for residents [15], blended learning for neurology postgraduates [16], and a randomised comparison of WeChat-based blended teaching versus traditional instruction for chamber operator trainees (*n* = 126) [32] reported improved examination scores, although most used single-group designs and the latter was published in a non-traditional indexed source.

Problem-based and case-based learning. A randomised trial of PBL teaching ward rounds reported significantly higher case analysis scores than traditional teaching (88.4 vs. 78.8, *p* < 0.05) among 32 trainees [17]. Descriptive studies of case-based teaching in hyperbaric neurology [18] and experiential teaching [19] described favourable learner experiences but provided no comparative outcome data.

Experiential, mentorship, and micro-teaching approaches. Descriptive reports of experiential teaching [19], humanised mentorship models [31,33], micro-teaching [34], elective curriculum design [41,42,46], and clinical teaching reports [38,43,44,45] collectively suggested favourable learner responses but lacked rigorous comparative evaluation.

Training needs and knowledge assessments. Across surveys and curriculum assessments, a consistent finding was a substantial gap in HBOT knowledge and training exposure among physicians, medical students, nurses, and residents [3,4,5,6,36,37], underscoring the unmet educational need that the limited intervention literature seeks to address.

### 3.5. Exploratory Quantitative Synthesis

A formal meta-analysis was not justified by the available data. Of the 30 included studies, only two reported sufficient quantitative information to compute a standardised effect size for a learner outcome. Zhang 2022 [17] (PBL versus traditional, case analysis score) yielded g = 2.11 (95% CI 1.24–2.98), and Paterni 2025 [14] (Web-based simulator, procedural performance, single-group) yielded a paired d = 2.36. These values are implausibly large and most plausibly reflect small-sample bias, absence of blinding, and practice or assessment effects rather than a genuine effect. The two randomised humanised mentorship trials assessed attitude/motivation and could not be pooled with these knowledge and procedural-outcome studies.No pooled estimate is therefore reported, and this exploratory synthesis of extractable effect sizes was not intended as a robustness test of any pooled estimate.

### 3.6. Learner Satisfaction

Across studies reporting satisfaction, technology-enhanced and simulation-based approaches were consistently rated as more engaging than traditional lectures, although this outcome was reported inconsistently and without standardised instruments.

## 4. Discussion

This systematic review identified 30 studies of HBOT education across eight databases and two languages. The central finding is sobering: despite clinical importance and a professed commitment to competency-based education, the evidence base for how best to teach HBOT is thin, methodologically weak, and dominated by descriptive or single-group studies. Only four randomised trials exist, and no multi-centre or adequately powered comparative study has been published.

### 4.1. Comparison with Prior Evidence

Our findings extend the prior systematic review by Boet et al. (2019), which found no comparative simulation studies in HBOT education [11]. Nearly two decades later, the number of comparative studies remains small, and the emergence of technology-enhanced modalities (Web simulators, VR, blended learning, scenario-based drills) has occurred almost entirely in single-group or quasi-experimental designs concentrated in Chinese-language journals. The field has expanded in breadth but not in methodological rigour.

### 4.2. Evidence Quality and the Problem of Inflated Effect Sizes

The exploratory quantitative synthesis provided a further, cautionary insight. The only two studies with extractable learner-outcome data reported standardised effect sizes exceeding 2.0—several-fold larger than the g = 0.3–0.8 typically observed for technology-enhanced teaching in the wider medical education literature [47,48,49]. Effect sizes of this magnitude are almost never reproduced in adequately powered, blinded trials and should be regarded as artefacts of small-sample bias, absence of blinding, and practice effects. This finding underscores that the HBOT education evidence base is not merely limited in volume but also at substantial risk of bias, reinforcing the need for caution in interpreting any individual positive study.

### 4.3. Implications for Curriculum Design

Notwithstanding the limitations, a consistent signal emerges: traditional didactic instruction alone is insufficient for the procedural and crisis management competencies central to safe HBOT practice, whereas simulation, blended learning, Web-based tools, and scenario-based drills appear to improve engagement and practical skills. We cautiously propose a staged, multi-modal framework—foundational knowledge via blended/online learning, procedural competence via simulation- and Web-based rehearsal, and crisis management via in situ simulation and emergency preparedness drills—while emphasising that this recommendation rests on limited, largely non-randomised evidence. Table S3 summarizes the best-evidence recommendations for HBOT curriculum design.

### 4.4. Limitations

First, the marked heterogeneity in study designs, learner populations, and outcome instruments precluded a quantitative meta-analysis, so conclusions rest on a narrative synthesis. Second, the methodological quality of the included studies was generally low: most used descriptive, survey, or single-group pre–post designs, and only four randomised trials were identified, most of which were small and at some concerns of bias. Third, the predominance of Chinese-language studies, many published in specialised or non-indexed journals, may limit the generalisability of our findings to other educational and healthcare systems. Fourth, publication bias could not be formally assessed given the small number of comparative studies; the inflated effect sizes in the two extractable studies are therefore interpreted as likely artefacts of small-sample bias, absence of blinding, and practice effects, rather than as evidence of publication bias per se. Fifth, the absence of a validated, standardised HBOT knowledge and skills assessment and the implausible magnitude of the few extractable effect sizes mean that the direction, rather than the magnitude, of reported effects should be interpreted with caution.

### 4.5. Future Research Directions

We identify five priorities: (1) adequately powered randomised trials with allocation concealment and blinded outcome assessment; (2) development and psychometric validation of a standardised HBOT knowledge and skills assessment; (3) a Delphi consensus to establish a core competency framework; (4) head-to-head trials comparing emerging technologies (VR versus Web-based simulation versus blended approaches); and (5) longitudinal studies linking educational outcomes to clinical safety metrics.

## 5. Conclusions

Technology-enhanced teaching modalities—particularly simulation-based education, scenario-based drills, blended learning, and Web-based simulation—are promising for HBOT education and appear to improve engagement and practical competence in the limited available evidence. However, the field lacks methodologically rigorous comparative studies: only four randomised trials exist, most included studies are descriptive, single-group or at high risk of bias, and no study yet demonstrates consistent superiority over traditional didactic teaching. Standardised outcome measures and a consensus-derived competency framework are urgently needed to advance HBOT education and translate the growing research base into improved training quality and patient safety.

## Figures and Tables

**Table 1:** Characteristics of included studies.

Study	Design	Learners	N	Intervention	Comparator	Reported Outcome/Focus
Sallusti 2001 [40]	Descriptive	Residents	NR	Theoretical + practical HBOT training	None	Programme description
Sadler 2019 [10]	Descriptive	Fellows/team	NR	Simulation curriculum (CNS O_2_ toxicity, ICU)	None	Curriculum design
Paganini 2021 [13]	Descriptive	Master’s students	NR	In situ simulation (8 scenarios)	None	Training method
Paterni 2025 [14]	Pre–post	Trainees	27	Web-based chamber simulator	Baseline	Knowledge gain
Talbot 2023 [3]	Survey	UGME curricula	7/17 schools	Curriculum audit	None	Curricular exposure
Jones 2017 [4]	Survey	Physicians	62	Knowledge assessment	None	Knowledge gaps
Magri 2020 [5]	Survey	Physicians	NR	Awareness survey	None	Awareness
Henrykowska 2018 [6]	Survey	Med students	240	Knowledge survey	None	Knowledge
Baines 2015 [37]	Survey	HBOT nurses	49	Workforce capability survey	None	Capability profile
Zhang 2021 [15]	Pre–post	Residents	65	WeChat blended learning	Baseline	Exam scores
Ren 2024 [16]	Pre–post	Postgrads	15	Blended learning (40 h)	Baseline	Exam
Zhang 2022 [17]	RCT	Trainees	32	PBL teaching ward rounds	Traditional	Case analysis
Wang 2023 [20]	Q-E	Aerospace students	29	VR (CAVE) + online cases	Lecture + visit	Knowledge
Zhang X 2024 [19]	Descriptive	Beginners	NR	Experiential teaching	None	Experience
Yu 2014 [18]	Descriptive	Neurology postgrads	NR	Case-based learning	None	Experience
Yang 2017 [31]	RCT	Trainees	70	Humanised mentorship	Traditional	Scores
Lyu 2015 [34]	Pre–post	Nurses	NR	Micro-teaching	Baseline	Skills
Hou 2017 [38]	Descriptive	Postgrads	NR	Clinical teaching	None	Experience
Tang 2012 [33]	RCT	Interns/trainees	70	Humanised mentorship	Traditional	Scores
Li 2009 [39]	Survey	Undergrads	200	Elective course survey	None	Cognition
Liang 2016 [45]	Descriptive	NR	NR	Teaching standardisation	None	Experience
Hao 2007 [46]	Descriptive	Undergrads	NR	Curriculum design	None	Curriculum proposal
Zhang 2023 [36]	Survey	Rotating trainees	214	Training needs survey	None	Needs assessment
Wang Q 2012 [41]	Descriptive	Undergrads	NR	Elective course standardisation	None	Experience
Bao 2017 [42]	Descriptive	Undergrads	NR	Heuristic + experiential teaching	None	Experience
Cheng 2006 [43]	Descriptive	Undergrads	NR	Clinical internship lecture	None	Experience
Dong 2015 [44]	Descriptive	Nurses	NR	Infant HBOT nursing teaching	None	Experience
Hu 2015 [21]	Pre–post	HBOT staff	NR	Scenario simulation drills	Baseline	Emergency response
Zhong 2020 [35]	Pre–post	HBOT nurses	NR	Scenario emergency drills	Baseline	Comprehensive ability
Li H 2021 [32]	RCT	Chamber operators	126	WeChat blended teaching	Traditional	Exam scores

Design: RCT = randomised controlled trial; Q-E = quasi-experimental; Pre–post = single-group before–after; Descriptive = curriculum/experience report; Survey = cross-sectional. NR = not reported.

## Data Availability

No new data were created or analyzed in this study. Data sharing is not applicable to this article.

## Supplementary Materials

The following supporting information can be downloaded at: https://www.mdpi.com/article/10.3390/healthcare14213661/s1, Table S1: Narrative synthesis by teaching modality; Table S2: Summary of included studies by learner level; Table S3: Best-evidence recommendations for HBOT curriculum design; Table S4: Per-study risk-of-bias assessment.

## References

[1] Moon R.E. (2019). Hyperbaric Oxygen Therapy Indications.

[2] Mathieu D., Marroni A., Kot J. (2017). Tenth European Consensus Conference on Hyperbaric Medicine: Recommendations for accepted and non-accepted clinical indications and practice of hyperbaric oxygen treatment. Diving Hyperb. Med..

[3] Talbot Z., Lee A., Boet S. (2023). Hyperbaric medicine in Canadian undergraduate medical school curriculum. Diving Hyperb. Med..

[4] Jones M.W. (2017). A pilot study evaluating knowledge of indications for hyperbaric oxygen therapy. Undersea Hyperb. Med..

[5] Magri K., Bigeni S., Azzopardi C.P., Camilleri L., Matity L., Muscat S., Meintjes W.A.J. (2020). Hyperbaric oxygen therapy awareness within a doctor population. Undersea Hyperb. Med..

[6] Henrykowska G., Buczyński A., Lewicka M., Zawadzka M. (2018). The knowledge of a selected group of medical students on hyperbaric oxygen therapy. Pol. Hyperb. Res..

[7] Leach R.M., Rees P.J., Wilmshurst P. (1998). Hyperbaric oxygen therapy. BMJ.

[8] Jain K.K. (2017). Textbook of Hyperbaric Medicine.

[9] Frank J.R., Snell L.S., Cate O.T., Holmboe E.S., Carraccio C., Swing S.R., Harris P., Glasgow N.J., Campbell C., Dath D. (2010). Competency-based medical education: Theory to practice. Med. Teach..

[10] Sadler C., Arefieva C.A.L., Duchnick J., Bermudez R., Witucki P., Shishlov K. (2019). Hyperbaric medicine simulation education curriculum: CNS toxicity during Treatment Table 6 and intubated ICU patient with mucous plugging. Undersea Hyperb. Med..

[11] Boet S., Cheng-Boivin O., Martin L., Hurskainen T., Etherington C. (2019). Evidence for simulation-based education in hyperbaric medicine: A systematic review. Diving Hyperb. Med..

[12] Boet S., Burns J.K., Jenisset E., Papp M., Bourbonnais S., Pignel R. (2022). A Delphi study to identify relevant scenarios as the first step toward an international hyperbaric medicine simulation curriculum. Diving Hyperb. Med..

[13] Paganini M., Mormando G., Savino S., Garetto G., Tiozzo G., Camporesi E.M., Fabris F., Bosco G. (2021). Emergency medicine cases in underwater and hyperbaric environments: The use of in situ simulation as a learning technique. Front. Physiol..

[14] Paterni M., Mastorci F., Benvenuti C., Bufano P., Melissa E., Zanoletti M., Lionetti V., Laurino M. (2025). A web-based training simulator of clinical hyperbaric chamber. Sci. Rep..

[15] Zhang Y., Yang J., Ma L.L., Liu X.H. (2021). Practice of hyperbaric oxygen clinical blended teaching based on WeChat platform for residents. China Contin. Med. Educ..

[16] Ren Z.Q., Liu Y.L., Liu Y.F., Wang W.J., Wang C., Yu Q.H. (2024). Application of blended model in training hyperbaric oxygen for postgraduates major in neurology. Chin. J. Stroke.

[17] Zhang Y., Liu X.H., Gao Y., Liang F., Yang L., Yang J. (2022). Application of problem-based learning in hyperbaric oxygen teaching ward rounds. Chin. Med. Rec..

[18] Yu Q.H., Zhang H.X., Liu Y.L., Yu J.C., Xue L.B. (2014). Application of case-based learning in clinical internship of hyperbaric oxygen neurology. Chin. J. Stroke.

[19] Zhang X.L., Yan M.Y. (2024). Application of experiential teaching in hyperbaric oxygen medicine for beginners. Chin. Med. Rec..

[20] Wang W.L., Li Y., Li W.B., Zhang X., Han P.J., Bao J.X. (2023). Application of virtual reality technology and online case teaching in aerospace medicine practice courses. Heart J..

[21] Hu Q.X., Yu X.M., Zhang M.Y. (2015). Application of scenario simulation drills in emergency plan training of hyperbaric oxygen department. Gen. Pract. Med. Clin. Educ..

[22] Kot J., Desola J., Gata Simao A., Gough-Allen R., Houman R., Meliet J.L., Galland F., Mortensen C., Mueller P.H.J., Sippinen S. (2004). A European code of good practice for hyperbaric oxygen therapy. Int. Marit. Health.

[23] Kot J., Desola J., Lind F., Mueller P., Jansen E., Burman F. (2023). A European code of good practice for hyperbaric oxygen therapy—Review 2022. Diving Hyperb. Med..

[24] Serena T.E., Gelly H., Bohn G.A., Niezgoda J.A. (2014). The American College of Hyperbaric Medicine consensus statement on physician credentialing for hyperbaric oxygen therapy. Adv. Skin Wound Care.

[25] Siermontowski P., Janas B., Olszański R. (2017). Formal and professional requirements for the staff of hyperbaric centres. Part 1. Pol. Hyperb. Res..

[26] Page M.J., McKenzie J.E., Bossuyt P.M., Boutron I., Hoffmann T.C., Mulrow C.D., Shamseer L., Tetzlaff J.M., Akl E.A., Brennan S.E. (2021). The PRISMA 2020 statement: An updated guideline for reporting systematic reviews. BMJ.

[27] Campbell M., McKenzie J.E., Sowden A., Katikireddi S.V., Brennan S.E., Ellis S., Hartmann-Boyce J., Ryan R., Shepperd S., Thomas J. (2020). Synthesis without meta-analysis (SWiM) in systematic reviews: Reporting guideline. BMJ.

[28] Sterne J.A.C., Savović J., Page M.J., Elbers R.G., Blencowe N.S., Boutron I., Cates C.J., Cheng H.Y., Corbett M.S., Eldridge S.M. (2019). RoB 2: A revised tool for assessing risk of bias in randomised trials. BMJ.

[29] Sterne J.A.C., Hernán M.A., Reeves B.C., Savović J., Berkman N.D., Viswanathan M., Henry D., Altman D.G., Ansari M.T., Boutron I. (2016). ROBINS-I: A tool for assessing risk of bias in non-randomised studies of interventions. BMJ.

[30] Miller G.E. (1990). The assessment of clinical skills/competence/performance. Acad. Med..

[31] Yang M.P. (2017). Effect of humanised mentorship model on improving the motivation of trainees in hyperbaric oxygen department. China Health Ind..

[32] Li H., Pan S.Y., Ding J.Z., Bai X.D. (2021). Practice and exploration of blended teaching based on WeChat platform in hyperbaric oxygen medicine education. Chin. Sci-Tech J. Database (Full-Text) Med. Health.

[33] Tang X.F. (2012). Effect of humanised mentorship model on improving internship motivation of interns and trainees in hyperbaric oxygen department. Chin. J. Pract. Nurs..

[34] Lyu M. (2015). Application of micro-teaching in clinical teaching of trainee nurses in hyperbaric oxygen department. Chin. Gen. Pract. Nurs..

[35] Zhong Y.L. (2020). Effect of scenario emergency drills on comprehensive quality of nurses in hyperbaric oxygen department. Contemp. Nurse (Acad. Ed.).

[36] Zhang Y., Zhu W.Q., Nan D., Yang L., He Y.F., Yang J. (2023). A survey of cognition and training needs of hyperbaric oxygen medicine among 214 rotating physicians. Chin. Med. Rec..

[37] Baines C., Sykes P. (2015). Professional capability within the Australian hyperbaric nursing workforce. Aust. J. Adv. Nurs..

[38] Hou X.M., Zhang Y., Ma L.L., Wang C., Gao C.J., Li Z. (2017). Discussion on clinical teaching of postgraduate students in hyperbaric oxygen specialty. Chin. Med. Rec..

[39] Li N., Li Q.M., Liao Z.L., Xu H.P., Wang M., Xie Y. (2009). Investigation and analysis of cognition and teaching implementation of the elective course Hyperbaric Oxygen Medicine among 200 military medical university undergraduates. Chongqing Med..

[40] Sallusti R., Ferraù S., Lozano Valdes A., Gonzales C., Jónsson M., Gullo A. (2001). Education and training in hyperbaric medicine. The Icelandic experience. An example of international scientific cooperation. Minerva Anestesiol..

[41] Wang Q., Zheng S.G. (2012). Teaching practice and standardization construction of the elective course Hyperbaric Oxygen Medicine. Chin. J. Med. Educ. Explor..

[42] Bao J.X., Xie X.P., Zhang H.J., Li J.S., Chang Y.M. (2017). Teaching practice and reflection on Hyperbaric Oxygen Medicine for clinical medicine undergraduates. Chin. J. Med. Educ. Explor..

[43] Cheng O.M., Wu X.S., Yang Q., Yan Y. (2006). Promoting the development of hyperbaric oxygen medicine: An attempt to offer lectures during clinical internship. Northwest Med. Educ..

[44] Dong H.Q., He Q., Hu J. (2015). Clinical teaching research on hyperbaric oxygen therapy and nursing for infants and young children. Chin. Gen. Pract. Nurs..

[45] Liang F., Yang J., Gao C.J., Li Z., Yang L., Liu X.H. (2016). Standardization construction of hyperbaric oxygen medicine teaching. Chin. Med. Rec..

[46] Hao Y.T., Li N., Xu H.P., Wu X.D. (2007). Conception of offering hyperbaric oxygen medicine course in five-year clinical medicine program. Chin. J. Med. Educ..

[47] Cook D.A., Hatala R., Brydges R., Zendejas B., Szostek J.H., Wang A.T., Erwin P.J., Hanstra S.J. (2011). Technology-enhanced simulation for health professions education: A systematic review and meta-analysis. JAMA.

[48] Vallée A., Blacher J., Cariou A., Sorbets E. (2020). Blended learning compared to traditional learning in medical education: Systematic review and meta-analysis. J. Med. Internet Res..

[49] McGaghie W.C., Issenberg S.B., Cohen E.R., Barsuk J.H., Wayne D.B. (2011). Does simulation-based medical education with deliberate practice yield better results than traditional clinical education? A meta-analytic comparative review of the evidence. Acad. Med..

