# Supplementary material for: Teaching Modalities for Hyperbaric Oxygen Therapy Education: A Systematic Review and Narrative Synthesis
Source: Healthcare (Basel). 2026 Oct 28;14(21):3661. doi: 10.3390/healthcare14213661 (PMC13762591; doi:10.3390/healthcare14213661)
Supplement: Supplementary file 1 [file healthcare-14-03661-s001.zip › Table S1.pdf]

Table S1. Narrative synthesis by teaching modality.

Teaching modality	Studies (n)	Design quality	Direction of evidence
Simulation / in situ / scenario drills	5	Mostly descriptive / pre-post	Improved engagement, confidence, emergency response
Web-based / VR simulation	2	Pre-post / Q-E	Improved knowledge; promising but limited
Blended / online / WeChat learning	4	1 RCT, others pre-post	Improved exam scores; single-group predominance
PBL / CBL	2	1 RCT, 1 descriptive	RCT favoured PBL (88.4 vs 78.8)
Experiential / humanised / micro-teaching	7	2 RCT, others descriptive	Favourable but inconsistent reporting
Curriculum / survey / needs assessment	10	Descriptive / survey	Documented large knowledge gaps
