# Supplementary material for: Teaching Modalities for Hyperbaric Oxygen Therapy Education: A Systematic Review and Narrative Synthesis
Source: Healthcare (Basel). 2026 Oct 28;14(21):3661. doi: 10.3390/healthcare14213661 (PMC13762591; doi:10.3390/healthcare14213661)
Supplement: Supplementary file 1 [file healthcare-14-03661-s001.zip › Table S2.pdf]

Table S2. Summary of included studies by learner level.

Learner level	Studies (n)	Reported N (range)	Key modalities evaluated
Undergraduate medical students	9	38–240	Elective, survey, curriculum, VR
Residents / fellows / postgraduate trainees	10	15–126	Blended, PBL, mentorship, simulation
Nurses / chamber staff	6	NR–49	Micro-teaching, scenario drills, surveys
Physicians / multidisciplinary / other	5	27–62	Web simulator, in situ sim, primers
