# Supplementary material for: Teaching Modalities for Hyperbaric Oxygen Therapy Education: A Systematic Review and Narrative Synthesis
Source: Healthcare (Basel). 2026 Oct 28;14(21):3661. doi: 10.3390/healthcare14213661 (PMC13762591; doi:10.3390/healthcare14213661)
Supplement: Supplementary file 1 [file healthcare-14-03661-s001.zip › Table S3.pdf]

Table S3. Best-evidence recommendations for HBOT curriculum design.

Miller’s level	Recommended modality	Evidence strength	Rationale
Knows	Blended/online self-study	Weak	Consistent but single-group data
Knows how	CBL / PBL case discussion	Weak–Moderate	One RCT favoured PBL
Shows how	Simulation (high-fidelity / in situ)	Weak	Engagement and emergency-response gains
Shows how (adjunct)	Web-based / VR chamber rehearsal	Weak	Pre-post improvement
Does	Supervised clinical practice	Consensus	No empirical comparative evidence
