# Supplementary material for: Teaching Modalities for Hyperbaric Oxygen Therapy Education: A Systematic Review and Narrative Synthesis
Source: Healthcare (Basel). 2026 Oct 28;14(21):3661. doi: 10.3390/healthcare14213661 (PMC13762591; doi:10.3390/healthcare14213661)
Supplement: Supplementary file 1 [file healthcare-14-03661-s001.zip › Table S4.pdf]

Supplementary Materials

Supplementary Table S4. Per-study risk-of-bias assessment (all 30 included studies (Samson 2025, excluded as an opinion/CME primer, is shown for transparency) (Samson 2025, excluded as an opinion/CME primer, is shown for transparency))

Tool: RoB 2 for randomised trials; ROBINS-I for comparative non-randomised studies; MMAT/JBI for descriptive, cross-sectional and survey designs.

**Study**	**Design**	**Tool**	**Overall judgement**	**Key concerns**
Sallusti 2001	Descriptive	MMAT	Unclear/high	Reporting, selection
Sadler 2019	Descriptive	MMAT	Unclear	Reporting completeness
Paganini 2021	Descriptive	MMAT	Unclear	Reporting completeness
Paterni 2025	Pre-post	JBI	High	No control, confounding
Talbot 2023	Survey	MMAT	Unclear	Sampling, response rate
Jones 2017	Survey	MMAT	Unclear	Sampling
Magri 2020	Survey	MMAT	Unclear	Sampling
Henrykowska 2018	Survey	MMAT	Unclear	Sampling
Baines 2015	Survey	MMAT	Unclear	Sampling
Samson 2025	Narrative review	—	Excluded*	Opinion/CME primer
Zhang 2021	Pre-post	JBI	High	No control
Ren 2024	Pre-post	JBI	High	No control
Zhang 2022	RCT	RoB 2	Some concerns	Blinding
Wang 2023	Q-E	ROBINS-I	Serious	Confounding, aerospace setting
Zhang X 2024	Descriptive	MMAT	Unclear	Reporting
Yu 2014	Descriptive	MMAT	Unclear	Reporting, neurology setting

Yang 2017	RCT	RoB 2	Some concerns	Blinding
Lyu 2015	Pre-post	JBI	High	No control
Hou 2017	Descriptive	MMAT	Unclear	Reporting
Tang 2012	RCT	RoB 2	Some concerns	Blinding
Li 2009	Survey	MMAT	Unclear	Sampling
Liang 2016	Descriptive	MMAT	Unclear	Reporting
Hao 2007	Descriptive	MMAT	Unclear	Reporting
Zhang 2023	Survey	MMAT	Unclear	Sampling
Wang Q 2012	Descriptive	MMAT	Unclear	Reporting
Bao 2017	Descriptive	MMAT	Unclear	Reporting
Cheng 2006	Descriptive	MMAT	Unclear	Reporting
Dong 2015	Descriptive	MMAT	Unclear	Reporting
Hu 2015	Pre-post	JBI	High	No control
Zhong 2020	Pre-post	JBI	High	No control
Li H 2021	RCT	RoB 2	Some concerns	Blinding

*Samson 2025 (a narrative CME primer) was identified as an opinion/educational-review piece inconsistent with the stated exclusion of opinion pieces and was therefore excluded from the synthesis; it is reported here for transparency and removed from Table 1 in the revised manuscript.
